# Vasectomy for Housing With Recipient of Embryo Transfer in the Common Marmoset ( *Callithrix jacchus* )

**DOI:** 10.1111/jmp.70042

**Published:** 2025-11-09

**Authors:** So‐Min Lee, Heejong Eom, GeunYong Lee, Jong‐Man Kim, Hee‐Jin Jang, Dohyun Lee

**Affiliations:** ^1^ Non‐Clinical Evaluation Center Osong Medical Innovation Foundation Cheongju Korea

**Keywords:** assisted reproductive technology, ductus deferens, monkey, non‐human primate, vasal disruption

## Abstract

Two male marmosets underwent vasectomy without complications. Azoospermia was confirmed by computer‐assisted semen analysis (CASA) 5 weeks later. Both were co‐housed with embryo transfer recipient females 6 weeks after surgery. Vasectomy may be accompanied by post‐vasectomy semen analysis such as CASA, to ensure safe and reliable sterilization for research and captive breeding management in marmosets.

**Abbreviations**
AAALAC: Accreditation of the Laboratory Animal Care International
AUA: American Urological Association
CASA: computer‐assisted semen analysis
ET: embryo transfer
PVSA: post‐vasectomy semen analysis

## 1 Introduction

Embryo transfer (ET) in the common marmoset ( *Callithrix jacchus* ) has become an essential assisted reproductive technology in reproductive research, as the marmoset model provides more relevant insights into human embryological development than the mouse embryo due to similarities in embryonic disc formation, yolk sac development, and haemochorial placentation as observed in humans [1, 2]. To increase the conception rate in ET, it is recommended that the recipient female remain with a contracepted male [3] or that the male paired with the recipient female be transferred to a separate cage 7–12 days prior to ovulation and returned at least 8 days after ovulation [3]. In zoological settings, contraception is also employed as a management strategy to regulate breeding within a colony and to maintain a stable captive population [4], thereby mitigating overcrowding, preserving social structure, and protecting animal welfare. Among available options, vasectomy is often considered the most effective contraceptive technique for male marmosets because it involves a short operation time, carries a lower risk of severe complications [5], and has minimal impact on male behavior, as the testes are retained, unlike in castration which is the conventional neutering approach for animals [4]. Although azoospermia can be easily detected by microscopy, precise assessment of sperm quantity and motility must be verified using computer‐assisted semen analysis (CASA) after vasectomy. We used vasectomy to prevent recipient females, which were intended for ET, from being fertilized by co‐housed males. The sterilized males were confirmed to have achieved contraception through CASA analysis.

## 2 Case Report

All animals originated from and were housed at the Nonclinical Evaluation Center, Osong Medical Innovation Foundation (Cheongju, Korea). All animal procedures adhered to the guidelines of the Institute of Laboratory Animal Resources [6], with approval from the Institutional Animal Care and Use Committee (KBIO‐IACUC‐2022‐078‐6). The Osong Medical Innovation Foundation holds full accreditation from the Association for Assessment and Accreditation of Laboratory Animal Care International (AAALAC). Marmosets were kept in stainless steel living cages (45 × 60 × 60 cm) with wire mesh floors. Environmental conditions in the animal rooms were controlled at 27°C ± 2°C, 40% ± 10% relative humidity, and maintained on a 12‐h/12‐h light/dark cycle (illumination ≥ 500 Lux), with 10–15 air exchanges per hour. Environmental enrichment included wooden perches for locomotion and gouging and a platform for bedding, placed in each cage. The animals were provided with a diet formulated for marmosets (50 g/day, No. 0630, Altromin, Lage, Germany), supplemented with vitamins and minerals, as well as ad libitum access to sterilized tap water.

Two adult male marmosets (ID: 325M and 490M), weighing 292 and 352 g and aged 7.5 years and 5.0 years, respectively, were included in this study. The animals underwent a 12‐h fasting period prior to surgery. Anesthesia was initiated with intramuscular injections (IM) of atropine (0.05 mg/kg), medetomidine hydrochloride (0.1 mg/kg), and ketamine (10 mg/kg). To maintain anesthesia, isoflurane (1.5%–2%) was administered by inhalation using a mask cone. Oxygen saturation and core body temperature were monitored continuously using a pulse oximeter placed on the hind limb paw and a rectal temperature probe, respectively. Body temperature was maintained at 38.5°C using a warming pad.

**FIGURE 1:**
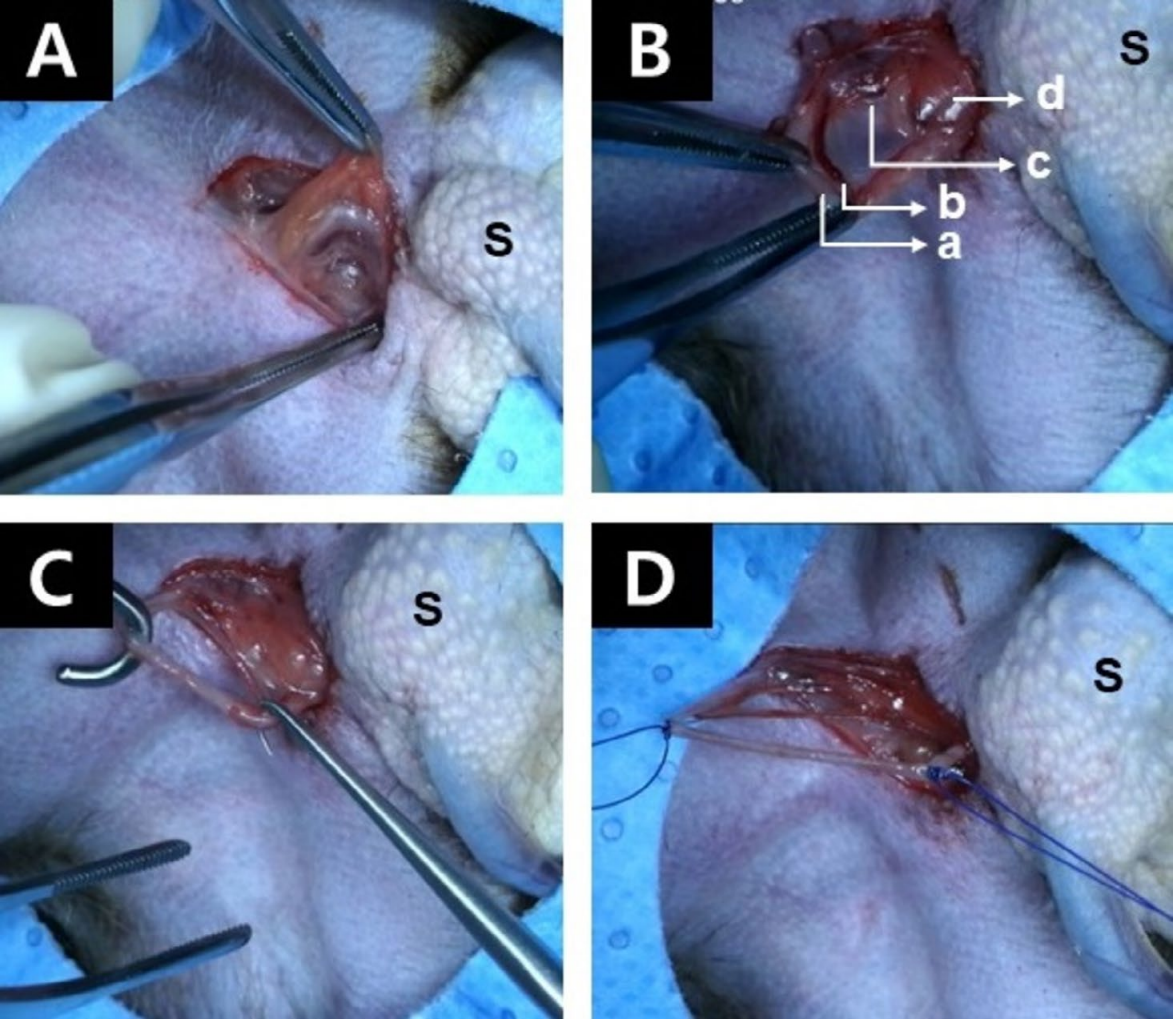
Identification of the complex formed by the ductus deferens, venous plexus, and testicular artery following blunt incision of the fascia (A). Elevation of the ductus deferens complex (a), testicular artery (b), and venous plexus (c) to expose a portion of testis (d) (B). The off‐white ductus deferens is elevated using a surgical hook, ensuring separation from the testicular artery and venous plexus (C). Both ends of the ductus deferens were tied with non‐absorbable suture and the segment between the ligations was excised (D). The left side of the figure indicates the direction of the head, and the right side indicates the direction where the scrotum (S) is located.

Vasectomy procedures were performed in accordance with American Urological Association (AUA) guidelines [5] and the methodology described in previous reports on marmosets [4]. The animal was placed in dorsal recumbency. The fur covering the surgical field, which extended from the ventral midline cranial to the genitals, was clipped. The area was then prepared aseptically for surgery. A round center‐cut drape was used to isolate the surgical site. An incision of 1.5 cm was made along the ventral midline at the pubic symphysis, extending 1 cm anterior to the cranial border of the scrotum. The fascia adjacent to the scrotum was bluntly dissected to expose the ductus deferens, venous plexus, and testicular artery, which were readily identifiable after blunt dissection (Figure 1A). The off‐white ductus deferens was carefully separated from the testicular artery and venous plexus and elevated using a surgical hook (Figure 1B,C). Both ends of the ductus deferens were ligated using non‐absorbable suture material (Dafilon; B. Braun, Melsungen, Germany), as absorbable sutures may result in recanalization and non‐absorbable suture is advised for vasectomy procedures [5]. The segment of the ductus deferens between the ligatures was excised (Figure 1D). The same procedure was performed on the contralateral ductus deferens. The subcutaneous tissue at the incision site was closed with a single mattress suture using polyglactin suture (Vicryl 4‐0; Ethicon, Raritan, NJ, USA). The edges of the skin incision were approximated and then sealed using surgical adhesive (Dermabond; Ethicon, Raritan, NJ, USA). The durations of the operations were 42 and 27 min for 490M and 325M, respectively. Following surgery, animals were placed in a warm recovery area and subsequently returned to their cages after full recovery. Postoperative care included administration of meloxicam (0.2 mg/kg, IM) and enrofloxacin (5 mg/kg, IM) daily for 3 days. Following surgery, vasectomized males were individually housed until azoospermia was confirmed, in order to ensure adequate recovery and to prevent any damage to the surgical site caused by other animals. Surgical sites were evaluated weekly for 4 weeks, and the wound margins had fully closed without complications by approximately 2–3 weeks following vasectomy.

Post‐vasectomy semen analysis (PVSA) was performed using a CASA system (SCA Production CASA system; Microptic, Barcelona, Spain) to quantitatively assess sperm concentration (million/mL) and motility parameters (total number and percentage), which were further categorized into progressive, motile and immotile sperm. Semen from male marmosets was collected for PVSA via penile vibrostimulation and processed in TYH medium (LSI Medience Corporation, Tokyo, Japan) to isolate spermatozoa [7]. In accordance with AUA guidelines, both manual and automated techniques, such as CASA, are considered appropriate for PVSA, contingent upon laboratory resources and proficiency [5]. Semen samples underwent evaluation using the SCA Production CASA system on the day of surgery, as well as at 3, 5, and 6 weeks following the procedure. The results of sperm concentration and motility are presented in Table 1. The initial total sperm concentrations were 18.61 and 24.79 million/mL (M/mL), which decreased to 6.32 and 1.27 M/mL, respectively, at 3 weeks post‐vasectomy. At 5 and 6 weeks post‐vasectomy, total sperm concentration was not detectable (0.00 M/mL). The proportion of motile sperm (calculated as a percentage of total sperm concentration) dropped from 7.62 to 10.14 M/mL (41% to 43%) at baseline to 0.03 to 0.07 M/mL (0.53% to 5.77%) at 3 weeks after vasectomy, and motile sperm were entirely absent (0.00 M/mL, 0%) at 5 and 6 weeks post‐vasectomy. Successful sterilization with confirmed azoospermia was verified at 5 weeks. Beginning 6 weeks after surgery, the animals were housed with female recipients for ET and did not demonstrate any atypical social behaviors, including deprivation, aggression, or refusal to partner for breeding [8] as observed over the 1‐month cohabitation period.

**TABLE 1:** Spermatozoa concentration (million/mL, M/mL) in semen from two marmosets (ID: 325M and 490M) before and after vasectomy.

Category	Before		Post 3‐week		Post 5‐week		Post 6‐week	
	325M	490M	325M	490M	325M	490M	325M	490M
Sperm concentration	18.61	24.79	6.32	1.27	0.00	0.00	0.00	0.00
Immotile	10.99 (59.08%)	14.05 (56.69%)	6.29 (99.47%)	1.20 (94.23%)	—	—	—	—
Motile	7.62 (40.92%)	10.14 (43.31%)	0.03 (0.53%)	0.07 (5.77%)	—	—	—	—
Progressive^a^	3.76 (20.21%)	5.45 (21.97%)	0.00 (0.00%)	0.00 (0.00%)	—	—	—	—
Non‐progressive^a^	3.86 (20.72%)	5.29 (21.34%)	0.03 (0.53%)	0.07 (5.77%)	—	—	—	—

*Note:* Total concentrations included both immotile and motile sperm, with motile sperm further categorized as progressive or non‐progressive; these sub‐classifications were displayed as a percentage of total sperm in parentheses. Concentrations were determined by post‐vasectomy semen analysis using computer‐assisted semen analysis.

^a^ Progressive indicates sperm that advance actively in either a straight or curved trajectory, while non‐progressive designates sperm that exhibit movement but lack linear progression, such as swirling or wobbling.

## 3 Discussion

Vasectomy was performed as a contraceptive method in two adult male marmosets that were to be housed with ET recipient females. The vasectomised animals did not exhibit any postoperative complications or abnormal behaviors, and they were housed with recipient females at 6 weeks post‐vasectomy after azoospermia was confirmed using the CASA system from 5 weeks post‐vasectomy. Vasectomy is considered the safest and most cost‐effective contraceptive option for males, and various techniques for vasal disruption have been developed to lower the failure rate in human medicine [9]. However, we chose the “ligation and excision” technique [10, 11] due to the size of the ductus deferens in marmosets, although the failure rate (1.5%–29.0%) associated with this method [10, 11] is slightly higher than that of other techniques such as “cautery and excision” (4.8% or less) [12], “cautery and fascial interposition” (1.2% or less) [12, 13], “ligation and fascial interposition” (16.7% or less) [11, 13], “intraluminal cautery” (< 1%) [14], and “cautery (open testicular end) and fascial interposition” (0.02% to 2.4%) [15, 16]. In this study, ligation of the ductus deferens was performed using non‐absorbable suture material because absorbable suture material can promote recanalization in marmosets [4].

Vasectomy in male non‐human primates (NHPs) has typically been performed as a means of population control in zoological settings [4, 17, 18]. However, it is relatively uncommon in laboratory NHPs, except in specific theriogenologic contexts, such as when males are housed with ET recipients [3]. The fundamental principle of vasectomy is to sever the ductus deferens and prevent their reconnection. In most vasectomy procedures for NHPs [17, 18], including marmosets [4]—as in the present study—the ductus deferens is ligated and excised. However, few studies have confirmed sterilization using reliable PVSA methods such as CASA, which was performed in this study. Vasectomy does not result in immediate sterility, and separation between male and female must be maintained until vas occlusion is verified by PVSA, typically performed between 8 and 16 weeks after vasectomy, as 80% of human males are azoospermic by 12 weeks post‐surgery [5]. In this study, PVSA was conducted in marmosets starting 3 weeks after vasectomy, and azoospermia was confirmed at 5 weeks. Marmosets demonstrated earlier onset of azoospermia compared to humans, possibly due to the spermatogenesis cycle of marmosets lasting 10 days relative to the 16‐day cycle in humans [19]. Additionally, no self‐mutilation was observed in vasectomized animals following skin closure with surgical adhesive, wounds healed successfully by approximately 2–3 weeks post‐surgery, and no further analgesia was necessary after treatments on post‐operative day 3.

## 4 Conclusion

Vasectomy is a safe sterilization method in males of NHP including marmosets, and it may be accompanied by PVSA such as CASA to secure reliable sterilization for research and captive breeding management in marmosets.

## Ethics Statement

All procedures were performed in compliance with the Institute of Laboratory Animal Resources guidelines [6] and were approved by the Institutional Animal Care and Use Committee (KBIO‐IACUC‐2022‐078‐6).

## Conflicts of Interest

The authors declare no conflicts of interest.

## Data Availability

The data that support the findings of this study are available from the corresponding author upon reasonable request.
